# An analysis of the relationship of triglyceride glucose index with diffuse large B-cell lymphoma prognosis: a retrospective study

**DOI:** 10.3389/fendo.2025.1573986

**Published:** 2025-04-28

**Authors:** QingQing Luo, Zhixiang Lei, Haizhou Miao, Ting Huang, Li Yu

**Affiliations:** ^1^ Department of Hematology, The Second Affiliated Hospital, Jiangxi Medical College, Nanchang University, Nanchang, Jiangxi, China; ^2^ School of Rehabilitation, Nanchang University, Nanchang, Jiangxi, China; ^3^ Department of Hematology, The Third People's Hospital of Pingxiang City, Pingxiang, Jiangxi, China

**Keywords:** diffuse large B-cell lymphoma, triglyceride-glucose index, insulin resistance, prognostic marker, nomogram

## Abstract

**Objective:**

Diffuse large B-cell lymphoma (DLBCL) as among the most common lymphomas is associated with insulin resistance (IR). The triglyceride-glucose (TyG) index, generally considered a surrogate marker for IR, has an uncertain prognostic value in DLBCL.

**Methods:**

We conducted a retrospective analysis of DLBCL patients who received R-CHOP therapy at the Second Affiliated Hospital of Nanchang University from January 2011 to December 2023. Univariate and multivariate Cox regression analyses were performed to identify independent prognostic factors for overall survival (OS). Boruta algorithm was employed to strengthen the robustness of our analysis. Restricted cubic spline (RCS) analysis was used to explore the potential nonlinear relationship between the TyG index and OS. Subgroup analyses were conducted to assess the prognostic value of the TyG index across different patient subgroups. Finally, a nomogram model based on the TyG index was developed, and its predictive performance was evaluated using the area under the receiver operating characteristic curve (AUROC), calibration curves, and decision curve analysis (DCA).

**Results:**

A total of 186 DLBCL patients were included in this study. Univariate and multivariate Cox regression analyses identified the TyG index, Age, ECOG performance status, Ann Arbor stage, and lactate dehydrogenase levels as independent prognostic factors for DLBCL. The Boruta algorithm confirmed these variables as the most important prognostic factors. Kaplan-Meier analysis revealed significantly poorer OS in the high TyG index group. RCS analysis demonstrated a non-linear relationship between the TyG index and OS. Subgroup analyses further validated the TyG index as a significant prognostic factor across various patient subgroups. The TyG-based nomogram model outperformed the conventional International Prognostic Index (IPI), with AUROCs of 0.878, 0.809, and 0.867 for 1-year, 3-year, and 5-year OS, respectively. Calibration curves showed good agreement between the nomogram predictions and actual outcomes, and DCA highlighted the high clinical utility of the model.

**Conclusion:**

The TyG index is an independent prognostic factor in DLBCL patients, and the TyG-based nomogram model provides enhanced predictive accuracy compared to the IPI. Its simplicity and low cost make it a valuable tool for routine clinical prognostic assessment in DLBCL patients.

## 1 Introduction

Diffuse large B-cell lymphoma (DLBCL) stands as the most frequent subset of non-Hodgkin lymphoma (NHL), comprising roughly 30% of all reported cases and exhibiting an annual incidence rate of 7 cases per 100,000 individuals, thereby posing a grave danger to human well-being (1). Despite improvements in overall survival (OS) with the R-CHOP regimen (rituximab in combination with cyclophosphamide, doxorubicin, vincristine, and prednisone), the 5-year survival rate remains only 60%-80% (2). The International Prognostic Index (IPI) is an essential tool for evaluating DLBCL prognosis, yet its predictive power has limitations in the rituximab era (3). While several novel prognostic markers have been identified, their clinical application is limited due to technical complexity and high costs (4). Therefore, there is a critical need to explore simple and accessible biomarkers to optimize existing prognostic evaluation systems.

Insulin resistance (IR) has been linked to the development and progression of several cancers, but the relationship with DLBCL is unclear (5, 6). Our previous research found that DLBCL patients had significantly lower levels of triglycerides (TG), high-density lipoprotein cholesterol (HDL-C), and low-density lipoprotein cholesterol (LDL-C) compared with healthy controls, with notable recovery following chemotherapy (7). This phenomenon may result from lipid metabolism disorders, which can regulate signaling pathways and alter the tumor microenvironment, ultimately contributing to the progression of DLBCL (8, 9). Additionally, DLBCL is characterized by elevated glycolytic activity, which fosters an immunosuppressive microenvironment, hindering antitumor immune responses (10). Increased glycolysis further affects systemic glucose metabolism, potentially worsening glycemic imbalances. Clinical studies have demonstrated that DLBCL patients with diabetes have significantly worse OS and progression-free survival (PFS) rates than non-diabetic patients, whereas those with effective blood glucose control during chemotherapy have better prognoses (11). These findings suggest that plasma metabolic markers associated with IR, such as glucose and lipids, are correlated with the prognosis of DLBCL.

The triglyceride-glucose (TyG) index, a recent focal point in medical research, is derived from triglyceride and glucose levels and has been validated as a reliable surrogate marker of IR, primarily associated with metabolic disorders and cardiovascular pathologies. A study by Fierro et al. (12) demonstrated that the TyG index is an independent risk factor for COVID-19-induced diabetes, while another study by Rabiee emphasized its predictive value for cardiovascular events (13). Furthermore, research by Zhu et al. (14) highlighted the association between the TyG index and the risk of non-alcoholic fatty liver disease, a condition closely linked to metabolic dysfunction. Moreover, emerging research have revealed that the TyG index is also linked to the development of various cancers, including breast cancer, prostate cancer, lung cancer, gastric carcinogenesis (15–18). The potential mechanisms underlying these relationships may include chronic inflammation caused by metabolic dysregulation, activation of the insulin-like growth factor axis, and metabolic changes in the tumor microenvironment (19). Although its relationship with DLBCL is not yet fully understood, existing evidence suggests an association between dysregulated glucose and lipid and DLBCL prognosis. Therefore, the TyG index holds potential as a prognostic marker in DLBCL.

The objective of this study is to investigate the correlation between the TyG index and the prognosis of DLBCL, aiming to evaluate its potential as a novel and readily accessible prognostic marker. To the best of our knowledge, this is the first study to explore this relationship. Moreover, we have developed a predictive model based on the TyG index, providing a tool for clinical decision-making in patients with DLBCL.

## 2 Patients and methods

### 2.1 Study subjects

**Figure 1:**
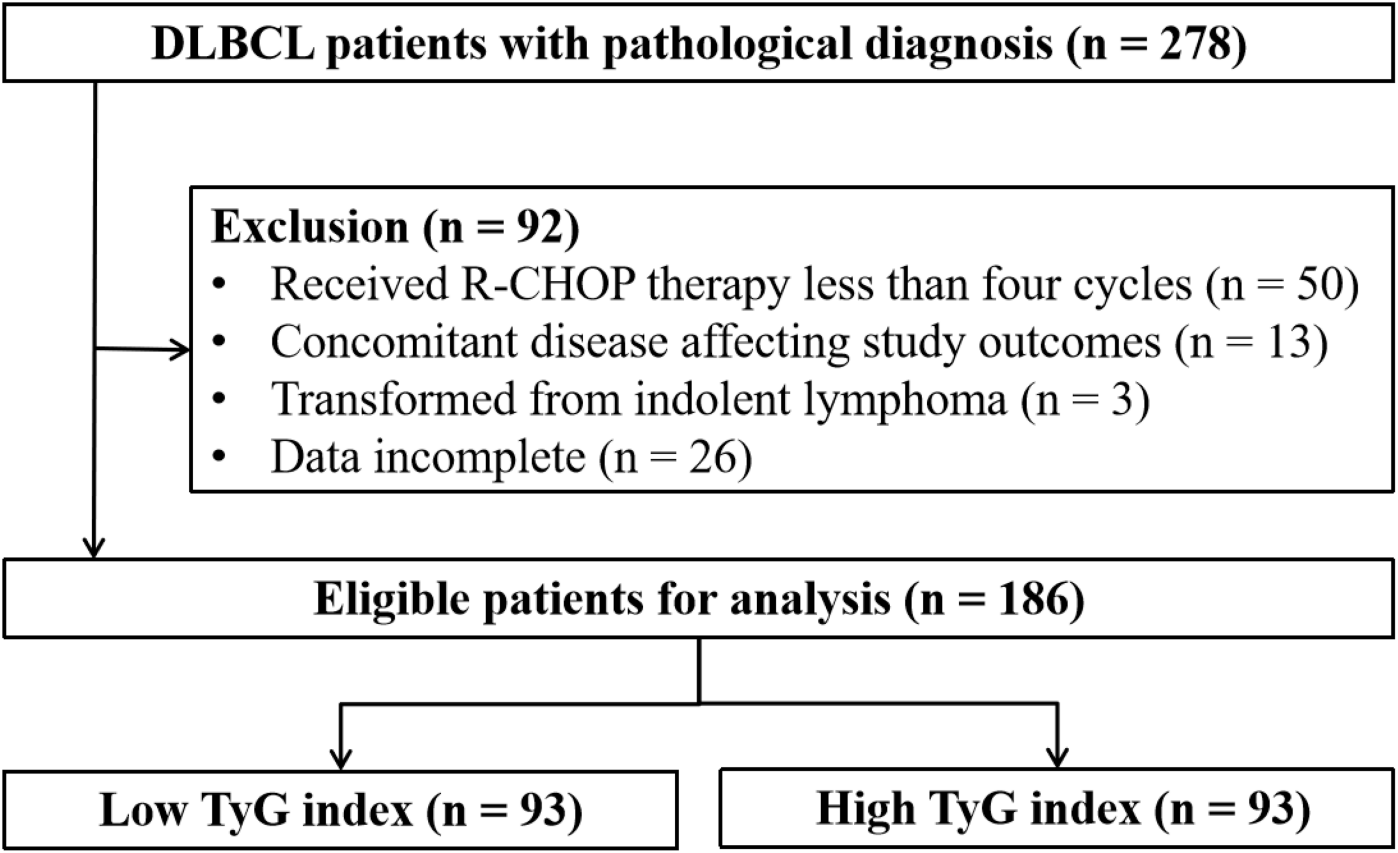
The flowchart of patient selection.

This retrospective study collected data from patients diagnosed with DLBCL at the Second Affiliated Hospital of Nanchang University between January 2011 and December 2023. Patients who were pathologically diagnosed for the first time were included in the study, but patients with the following were excluded (1): received R-CHOP therapy less than four cycles; (2) concomitant disease affecting study outcomes; (3) transformed indolent lymphomas; (4) missing essential laboratory data such as glucose and lipid levels. **Figure 1** shows the flowchart of patient selection.

### 2.2 Data collection

Baseline clinical data were collected from the electronic medical records system of the Second Affiliated Hospital of Nanchang University. The data included: (1) General information: Age, Sex, Body Mass Index (BMI), Ann Arbor stage, B symptoms, Eastern Cooperative Oncology Group Performance Status (ECOG PS), IPI, number and sites of extranodal involvement, and chemotherapy regimens. (2) Pathological data: Ki67 levels and Hans classification. All histopathological slides were reviewed independently by two pathologists to classify tumors as germinal center B-cell (GCB) or non-GCB types according to Hans criteria (20). (3) Laboratory data from the first examination before treatment: neutrophil count, lymphocyte count, platelet count, hemoglobin (Hb), albumin (ALB), lactate dehydrogenase (LDH), fasting blood glucose (FBG), triglycerides (TG), total cholesterol (TC), high-density lipoprotein cholesterol (HDL-C), low-density lipoprotein cholesterol (LDL-C), apolipoprotein A (APOA), apolipoprotein B (APOB) and inflammation-derived index: neutrophil-to-lymphocyte ratio (NLR), platelet-to-lymphocyte ratio (PLR), systemic immune-inflammation index (SII). The primary outcome of interest was OS, defined as the duration from treatment initiation to death from any cause.

### 2.3 TyG index assessment

The TyG index was calculated using the formula: TyG index = Ln [TG (mg/dL) × FBG (mg/dL)/2]. Based on a median TyG index of 1.30, patients were categorized into high and low TyG index groups. Univariate and multivariate Cox regression analyses were performed to identify independent prognostic factors affecting survival. In addition, these results were further validated using the Boruta algorithm, Kaplan-Meier (KM) curve survival analysis, and subgroup analysis. A restricted cubic spline (RCS) analysis was utilized to investigate the dose-response relationship between TyG index as a continuous variable and OS of DLBCL patients.

### 2.4 Establishment and evaluation of a prognostic model

Based on the identified independent prognostic factors, we used multivariate Cox regression analysis to construct a model for predicting survival in patients with DLBCL. To ensure the accuracy of the model, we performed proportional risk hypothesis testing and model impact point identification. To assess the predictive performance of the model, we used the receiver operating characteristic (ROC) curve and their area under the curve (AUC). In addition, we plotted calibration curves to verify the consistency of the predicted results with the actual results. Finally, through decision curve analysis (DCA), we further evaluated the value of the model in clinical practice.

### 2.5 Statistical methods

The data analysis was conducted using SPSS 25.0 statistical software (IBM, Chicago, USA). To compare differences in clinical indicators between the two groups, we employed either the Wilcoxon rank-sum test or Pearson’s chi-squared test. Kaplan-Meier survival analysis coupled with the log-rank test was utilized to assess the correlation between prognostic factors and survival outcomes. ROC curve analysis, calibration curve, and DCA were performed using R software, specifically with the “pROC”, “rms”, and “ggDCA” packages, respectively. P < 0.05 was considered statistically significant.

## 3 Results

### 3.1 Patient characteristics

Among the 186 patients included, ages at diagnosis ranged from 14 to 90 years, with 102 males (54.8%). A total of 56.5% were classified as Ann Arbor stage III-IV, 36.6% had elevated LDH levels, and 17.7% presented with B symptoms. By the end of the follow-up period (median: 36 months), 49 patients (26.3%) had died. **Table 1** provides the clinical and demographic characteristics of both groups, with statistically significant differences observed in BMI, FBG, TG, and ApoB levels (P < 0.05).

**Table 1:** Baseline characteristics of patients grouped by median TyG index.

Variables	TyG index			P value
	Overall, N = 186	Low, N = 93	High, N = 93	
Sex				0.377
Female	84 (45.2%)	45 (48.4%)	39 (41.9%)	
Male	102 (54.8%)	48 (51.6%)	54 (58.1%)	
Age (years)				0.141
≤60	100 (53.8%)	55 (59.1%)	45 (48.4%)	
>60	86 (46.2%)	38 (40.9%)	48 (51.6%)	
BMI	22.8 (21.1, 24.4)	22.8 (20.7, 23.2)	22.9 (21.5, 25.3)	0.015
NEUT (*10^9^/L)	3.86 (3.07, 4.97)	3.69 (2.87, 4.75)	3.88 (3.15, 4.99)	0.236
LYM (*10^9^/L)	1.20 (0.84, 1.69)	1.13 (0.79, 1.54)	1.27 (0.99, 1.79)	0.078
HB (g/L)	124 (109, 137)	121 (102, 136)	126 (112, 140)	0.080
PLT (*10^9^/L)	210 (168, 279)	210 (170, 290)	209 (167, 263)	0.482
ALB (g/L)	39.3 (38.3, 42.2)	39.3 (38.2, 40.9)	39.7 (38.5, 42.7)	0.056
FBG (mmol/L)	5.45 (4.79, 6.69)	5.15 (4.65, 5.61)	6.36 (5.24, 7.98)	<0.001
TG (mmol/L)	1.34 (0.96, 1.88)	1.00 (0.80, 1.22)	1.87 (1.48, 2.44)	<0.001
TC (mmol/L)	4.18 (3.58, 4.73)	4.13 (3.55, 4.61)	4.22 (3.66, 5.01)	0.126
HDL (mmol/L)	1.01 (0.82, 1.29)	1.05 (0.82, 1.36)	0.99 (0.82, 1.19)	0.069
LDL (mmol/L)	2.46 (2.07, 2.91)	2.45 (2.13, 2.88)	2.46 (2.04, 2.91)	0.880
ApoA (g/L)	1.09 (0.85, 1.40)	1.03 (0.78, 1.37)	1.14 (0.91, 1.40)	0.167
ApoB (g/L)	0.85 (0.70, 0.95)	0.82 (0.67, 0.91)	0.85 (0.76, 0.98)	0.009
LDH				0.761
Low	118 (63.4%)	60 (64.5%)	58 (62.4%)	
High	68 (36.6%)	33 (35.5%)	35 (37.6%)	
ECOG PS				0.203
Low	169 (90.9%)	87 (93.5%)	82 (88.2%)	
High	17 (9.1%)	6 (6.5%)	11 (11.8%)	
B symptoms				0.084
No	153 (82.3%)	81 (87.1%)	72 (77.4%)	
Yes	33 (17.7%)	12 (12.9%)	21 (22.6%)	
Hans				0.091
Non-GCB	121 (65.1%)	66 (71.0%)	55 (59.1%)	
GCB	65 (34.9%)	27 (29.0%)	38 (40.9%)	
Extranodal site				0.426
0	129 (69.4%)	67 (72.0%)	62 (66.7%)	
1	57 (30.6%)	26 (28.0%)	31 (33.3%)	
Ann Arbor stage				0.657
Low	81 (43.5%)	42 (45.2%)	39 (41.9%)	
High	105 (56.5%)	51 (54.8%)	54 (58.1%)	
IPI				0.875
0-2	127 (68.3%)	64 (68.8%)	63 (67.7%)	
3-5	59 (31.7%)	29 (31.2%)	30 (32.3%)	
Ki 67	0.73 (0.70, 0.80)	0.73 (0.70, 0.80)	0.80 (0.70, 0.80)	0.633
NLR	3.1 (2.0, 5.0)	3.1 (2.0, 5.0)	3.3 (2.0, 4.8)	0.862
PLR	182 (117, 280)	192 (117, 317)	175 (116, 247)	0.062
SII	654 (384, 1,280)	687 (360, 1,360)	645 (392, 1,164)	0.754

BMI, body mass index; NEUT, neutrophil count; LYM, lymphocyte count; HB, hemoglobin; PLT, platelet count; ALB, albumin; ECOG PS, eastern cooperative oncology group performance status; GCB, germinal center B-cell; IPI, international prognostic index; LDH, lactate dehydrogenase; FBG, fasting blood glucose; TG, triacylglycerol; TC, total cholesterol; HDL-C, high-density lipoprotein cholesterol; LDL-C, low-density lipoprotein cholesterol; ApoA, apolipoprotein A; ApoB, apolipoprotein B; TyG index, triglyceride glucose index; NLR, neutrophil to lymphocyte ratio; PLR, platelet to lymphocyte ratio; SII, systemic immune inflammation index.

### 3.2 Association between TyG index and prognosis of DLBCL patients

Univariate Cox regression analysis showed that Age, ECOG PS, B symptoms, Ann Arbor stage, extranodal involvement, LDH, TyG index, TC, HDL-C, and LDL-C were all associated with OS (P < 0.05). Multivariate Cox regression analysis further identified Age, ECOG PS, Ann Arbor stage, LDH, and TyG index as independent prognostic factors for OS (P < 0.05) (**Table 2**).

**Table 2:** Univariate and multivariate Cox hazards analysis for OS in DLBCL.

Vb Variables	Univariable		Multivariable	
	HR (95% CI)	P value	HR (95% CI)	P value
Sex				
Female	reference			
Male	1.45 (0.81-2.60)	0.208		
Age				
≤60	reference		reference	
>60	3.29 (1.77-6.12)	<0.001	3.50 (1.84-6.67)	<0.001
BMI	0.96 (0.87-1.06)	0.393		
NEUT (*10^9^/L)	1.02 (0.98-1.06)	0.303		
LYM (*10^9^/L)	1.14 (0.71-1.80)	0.591		
HB (g/L)	0.99 (0.98-1.00)	0.053		
PLT (*10^9^/L)	1.00 (1.00-1.00)	0.569		
ALB (g/L)	0.95 (0.89-1.02)	0.163		
FBG (mmol/L)	1.07 (0.97-1.18)	0.162		
TG (mmol/L)	1.10 (0.85-1.41)	0.472		
TC (mmol/L)	0.57 (0.41-0.79)	<0.001	0.46 (0.20-1.08)	0.074
HDL (mmol/L)	0.41 (0.19-0.89)	0.024	2.14 (0.84-5.47)	0.112
LDL (mmol/L)	0.51 (0.32-0.82)	0.005	1.32 (0.48-3.58)	0.590
ApoA (g/L)	0.62 (0.30-1.30)	0.208		
ApoB (g/L)	0.98 (0.28-3.43)	0.973		
LDH				
Low	reference		reference	
High	3.08 (1.74-5.45)	<0.001	2.04 (1.08-3.86)	0.028
ECOG PS				
Low	reference		reference	
High	4.53 (2.31-8.91)	<0.001	3.16 (1.48-6.72)	0.003
B symptoms				
No	reference		reference	
Yes	2.65 (1.44-4.89)	0.002	1.51 (0.77-2.98)	0.230
Extranodal site				
0	reference		reference	
1	3.03 (1.72-5.35)	<0.001	1.41 (0.72-2.74)	0.318
Hans				
Non-GCB	reference			
GCB	1.75 (1.00-3.06)	0.052		
Ann Arbor stage				
Low	reference		reference	
High	4.43 (2.15-9.14)	<0.001	2.55 (1.12-5.79)	0.026
Ki 67	1.08 (0.14-8.46)	0.939		
NLR	1.00 (0.99-1.02)	0.459		
PLR	1.00 (1.00-1.00)	0.588		
SII	1.00 (1.00-1.00)	0.378		
TyG index				
Low	reference		reference	
High	2.22 (1.22-4.03)	0.009	2.57 (1.25-5.31)	0.011

BMI, body mass index; NEUT, neutrophil count; LYM, lymphocyte count; HB, hemoglobin; PLT, platelet count; ALB, albumin; ECOG PS, eastern cooperative oncology group performance status; GCB, germinal center B-cell; IPI, international prognostic index; LDH, lactate dehydrogenase; FBG, fasting blood glucose; TG, triacylglycerol; TC, total cholesterol; HDL-C, high-density lipoprotein cholesterol; LDL-C, low-density lipoprotein cholesterol; ApoA, apolipoprotein A; ApoB, apolipoprotein B; TyG index, triglyceride glucose index; NLR, neutrophil to lymphocyte ratio; PLR, platelet to lymphocyte ratio; SII, systemic immune inflammation index.

**Figure 2:**
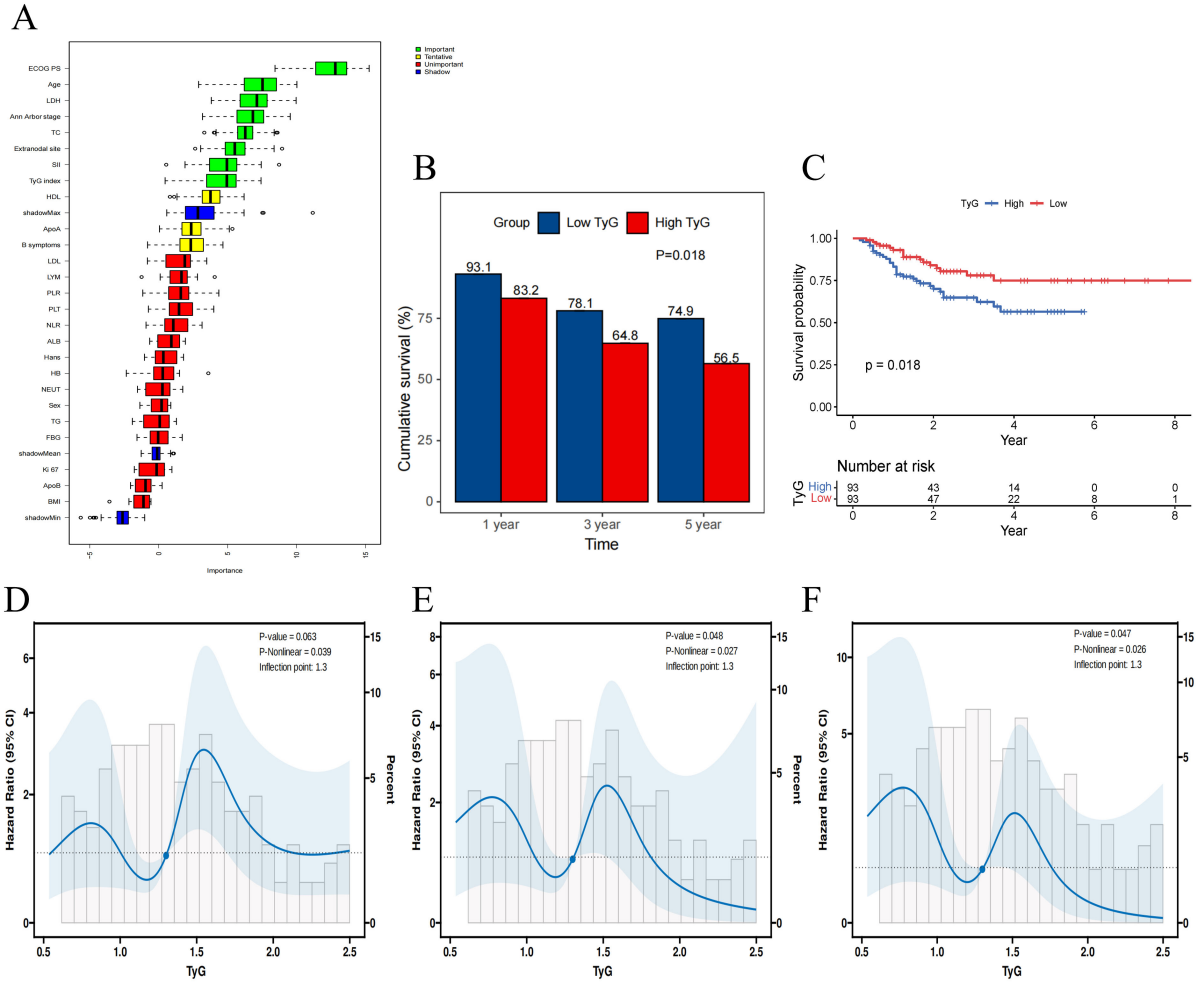
The association between TyG index and overall survival. **(A)** Importance ranking of variables affecting overall survival in Boruta analysis. **(B)** The 1-, 3-, and 5- year OS rates for high and low TyG Index groups. **(C)** Kaplan-Meier survival curves for overall survival in high and low TyG index groups. **(D-F)** Restricted cubic spline regression analysis of the TyG index for overall survival in patients with DLBCL. **(D)** unadjusted; **(E)** adjusted for TG and FBG; **(F)** adjusted for TG, FBG, BMI, ApoB.

The Boruta feature selection algorithm further confirmed the importance of these five variables in DLBCL prognosis (**Figure 2A**). As shown in **Figure 2B**, the 1-, 3-, and 5- year OS rates was higher in the patient group with low TyG index than in the patient group with high TyG index, with values of 93.1% *vs*. 83.2%, 78.1% *vs.* 64.8%, and 74.9% *vs.* 56.5%, respectively. The Kaplan-Meier survival analyses further demonstrated that the TyG index of the high patient group had a significantly lower OS than the patient group with a low TyG index (P = 0.018), and this difference was statistically significant (**Figure 2C**).

RCS analysis was used to determine whether there was a potential linear or nonlinear association between TyG index and OS in patients with DLBCL. The results indicated that TyG index was nonlinearly associated with OS in DLBCL (P-overall = 0.063, P-nonlinear = 0.039). After further adjustment for covariates, there was still a nonlinear relationship between the TyG index and OS in DLBCL patients in both the partially adjusted model (P-overall = 0.048, P-nonlinear = 0.027) and the fully adjusted model (P -overall = 0.047, P-nonlinear = 0.026) (**Figures 2D–F**).

### 3.3 Subgroup analysis

**Figure 3:**
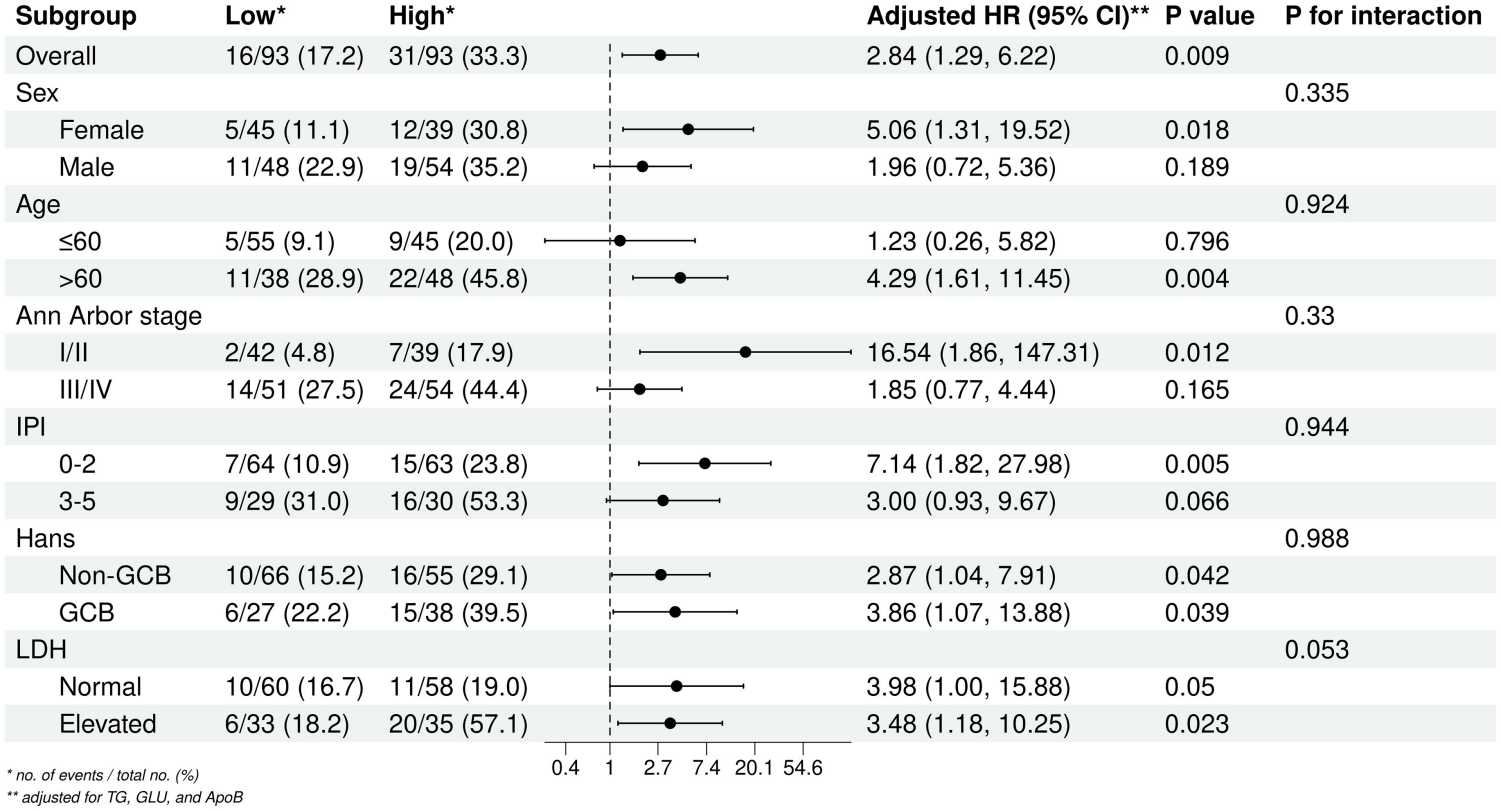
Subgroup analyses of the association between the TyG index and DLBCL prognosis.

To further examine the relationship between the TyG index and DLBCL prognosis, subgroup and interaction analyses were conducted across different Age, Sex, Ann Arbor stage, IPI, Hans classification, and LDH levels groups (**Figure 3**). The results indicated that a high TyG index is a prognostic risk factor for patients aged > 60 years, Ann Arbor stage III/IV, and IPI 0-2 group. The TyG index remained significantly associated with prognosis regardless of Hans classification or LDH levels. No significant interactions were observed across subgroups.

### 3.4 A prognostic model based on the TyG index

**Figure 4:**
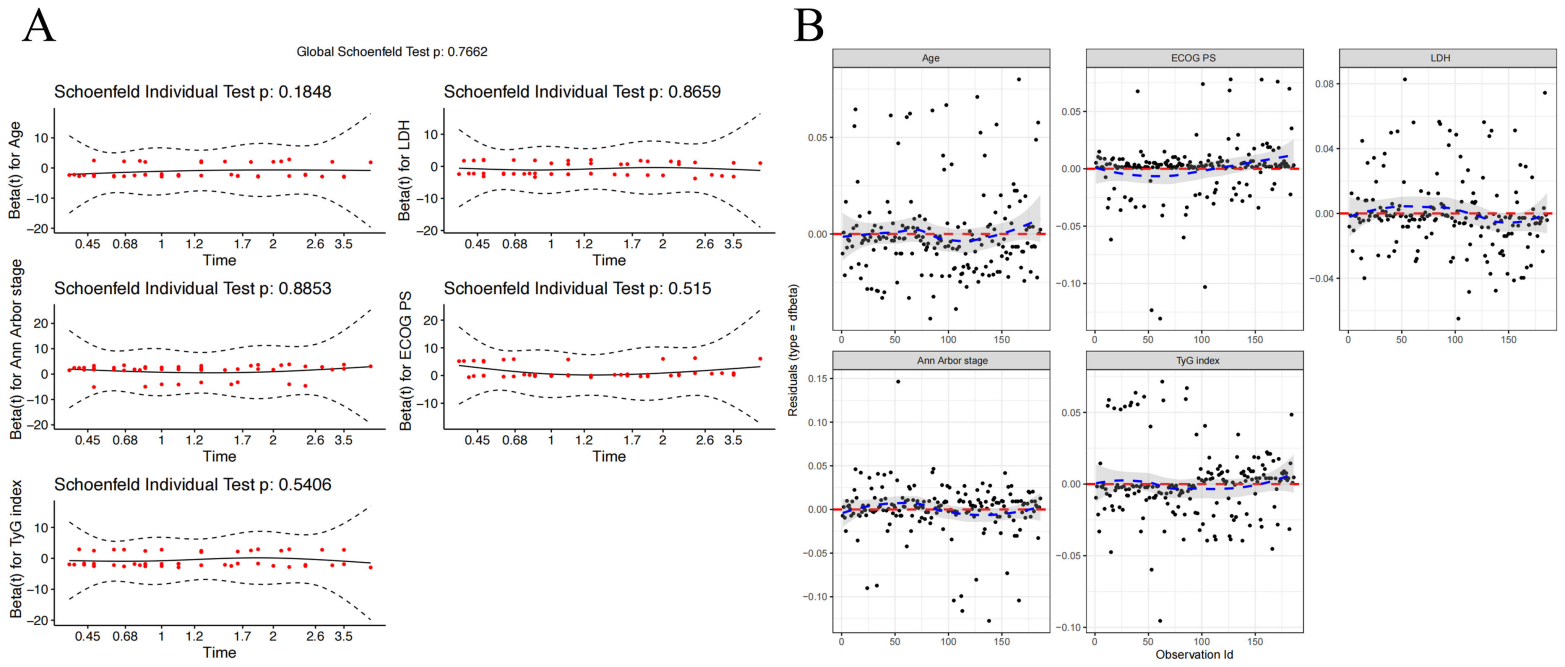
Diagnostics of the Cox Proportional Hazards Model. **(A)** Schoenfeld Residuals Plot for Age, ECOG PS, LDH Level, Ann Arbor Stage, and TyG Index. **(B)** Dfbeta Values for Age, ECOG PS, LDH Level, Ann Arbor Stage, and TyG Index.

Using Age, ECOG PS, LDH level, Ann Arbor stage, and TyG index, we constructed a model to predict 1-, 3-, and 5-year OS for DLBCL patients. To ensure the accuracy and robustness of the model, we performed proportional hazards assumption testing (PH test) and identified influence points within the model. The results of the PH test indicated that all five variables met the proportional hazards assumption (**Figure 4A**). By plotting Dfbeta values, we examined the impact of each observation on the model results (**Figure 4B**). The results show that even though some of the dfbeta values are very large, they are not large enough to have an impact on the estimates of the model coefficients.

**Figure 5:**
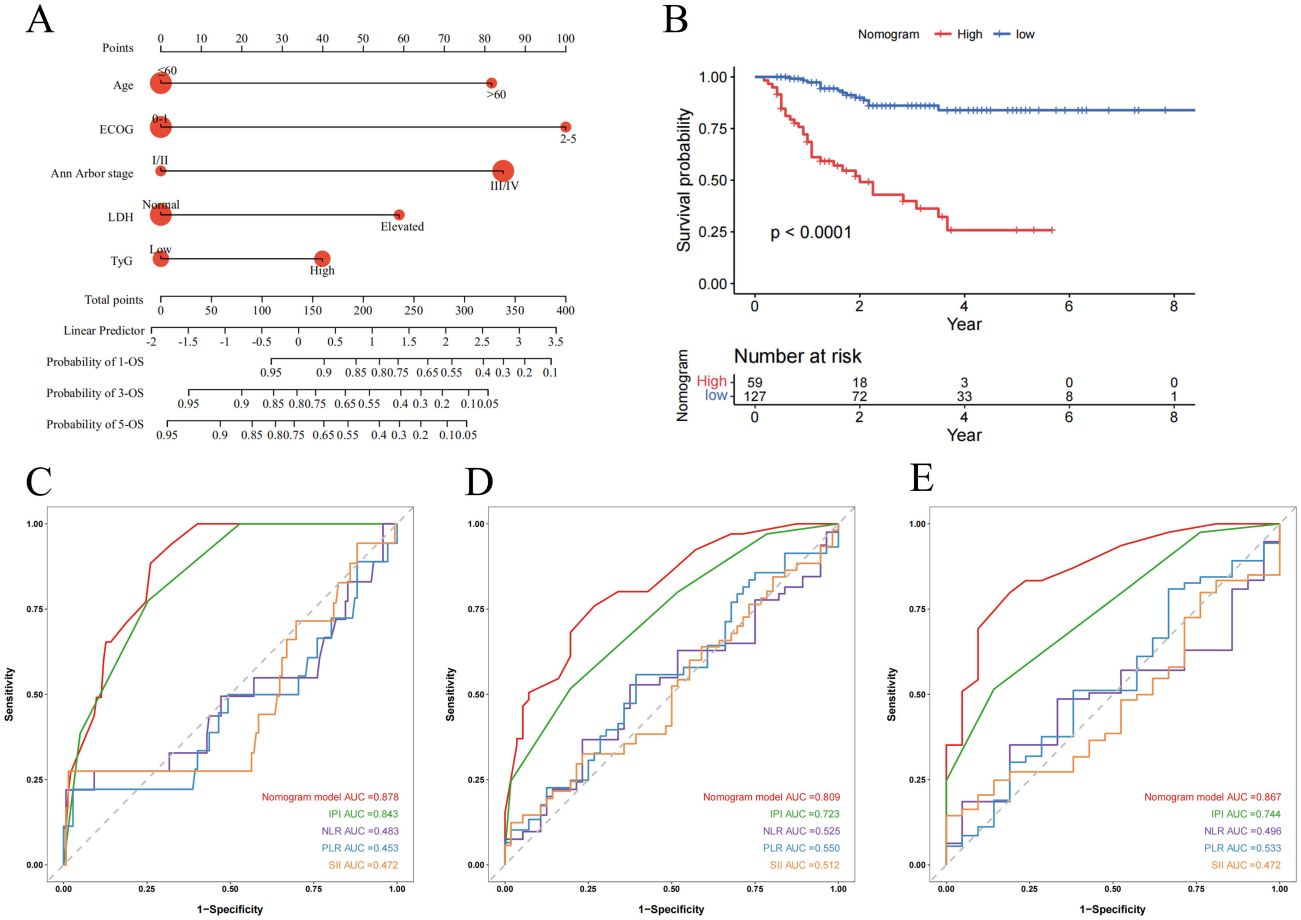
Nomogram and its receiver operating characteristic curves for predicting 1-, 3-, and 5-year overall survival. **(A)** Nomogram for predicting 1-, 3-, and 5-year OS; **(B)** Kaplan-Meier curves for OS in DLBCL patients stratified by risk category; **(C-E)** Receiver operating characteristic curves and area under the curve (AUC) for predicting 1-, 3-, and 5-year OS of the nomogram model compared with IPI, NLR, PLR and SII.

The nomogram for this model is visually presented in **Figure 5A**. Based on the optimal cutoff value of 0.415, patients were categorized into high-risk and low-risk groups. Kaplan-Meier survival analysis demonstrated that high-risk patients had significantly poorer OS compared to low-risk patients (P < 0.0001) (**Figure 5B**). The predictive performance of the nomogram was significantly better than that of IPI, NLR, PLR and SII, with AUCs of 1-, 3-, and 5-year OS of 0.878 (95% CI: 0.815-0.940), 0.809 (95% CI: 0.722-0.896), and 0.867 (95% CI: 0.777-0.956), respectively (**Figures 5C–E**).

**Figure 6:**
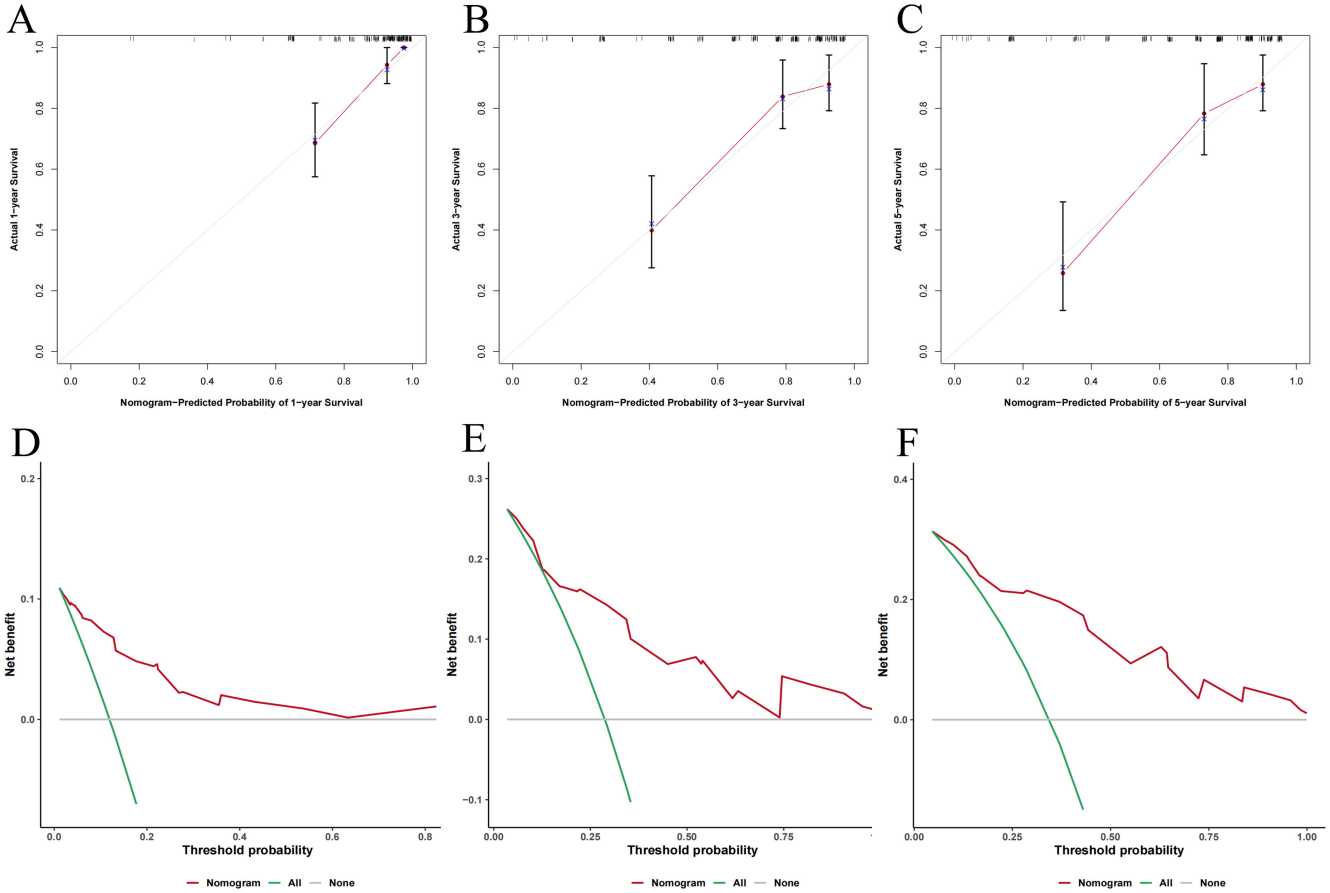
The predictive performance of nomogram model. **(A-C)** Calibration curves for 1-, 3-, and 5-year OS; **(D-F)** Decision curve analysis for 1-, 3-, and 5-year OS.

The 1-, 3-, and 5-year calibration curves indicated good agreement between the nomogram’s predictions and actual outcomes (**Figures 6A–C**). DCA showed higher net benefits across various threshold probabilities, supporting the model’s high clinical utility (**Figures 6D–F**).

## 4 Discussion

DLBCL is a highly heterogeneous malignancy with generally poor prognosis. The traditional IPI score has limitations in predicting the prognosis of DLBCL, highlighting the need for more precise prognostic tools. This study is the first to investigate the association between the TyG index and DLBCL prognosis. Our results identified the TyG index as an independent risk factor for DLBCL, with significantly higher survival rates in the low TyG group compared to the high TyG group (p < 0.05). Moreover, the TyG index remained correlated with poor prognosis across different patient subgroups. Multivariate Cox regression analysis revealed age, ECOG score, Ann Arbor stage, LDH levels, and the TyG index as independent prognostic factors influencing OS. Using these five indicators, we developed a nomogram model that surpasses the traditional IPI in predictive performance, with the TyG index as the only substitution. Calibration and decision curve analyses further confirmed the high predictive accuracy and clinical utility of this TyG-based model. Therefore, the TyG index may serve as a potential prognostic marker for DLBCL, and the TyG-based model provides a more reliable tool for assessing DLBCL prognosis.

The TyG index, a novel marker combining glucose and triglycerides, has been shown to be associated with the occurrence of various cancers. In a study by Li et al. (21) involving 767 participants, including 136 prostate cancer (PCa) patients and 631 healthy controls, the TyG index was identified as an independent risk factor for PCa. Similarly, Zhang et al. (15) found that breast cancer (BC) patients had significantly higher TyG index compared with benign breast disease, and that BC risk was positively correlated with rising TyG index levels. The TyG index was also associated with the occurrence of non-small cell lung cancer, colorectal adenoma, and gastric carcinogenesis (17, 18, 22). Moreover, the TyG index affects cancer patient outcomes, although research on this correlation remains limited. Fritz et al. (16) followed 259,884 men across eight European cohorts and found a positive correlation between the TyG index and PCa-specific mortality. Another study of 3,524,459 cancer patients showed that the TyG index had an inverse L-shaped relationship with all-cause and cardiovascular mortality (23). These findings suggest that the TyG index is a risk factor in cancer onset and progression, consistent with our results.

Existing research has confirmed that IR is a significant risk factor for the development and poor prognosis of lymphoma (24). However, the exact mechanisms by which IR directly impacts lymphoma remain unclear. Notably, certain potential mechanisms related to IR and its associated metabolic abnormalities (such as hyperinsulinemia) may offer crucial clues for understanding this association. Firstly, IR is considered a key factor in cell proliferation. Studies have shown that hyperinsulinaemia may affect energy metabolism by increasing cellular uptake of glucose, which activates certain intracellular signaling pathways, thereby enhancing cell proliferation and inhibiting apoptosis, and ultimately contributing to the development of cancer (19). In addition, insulin may promote malignant transformation, cancer progression and metastasis through binding and activation of the IGF-1 receptor, and hyperglycaemia itself may increase cellular sensitivity to IGF-1, further contributing to cancer development and progression (25, 26). Furthermore, IR is capable of triggering oxidative stress and inflammatory responses. It may lead to overproduction of reactive oxygen species (ROS), which in turn causes oxidative DNA damage and somatic mutations, thereby increasing the risk of tumorigenesis. Studies have revealed that IR indirectly promotes tumor growth through pro-inflammatory signaling pathways such as NF-κB. Abnormally elevated blood glucose can also exacerbate oxidative stress and drives chronic inflammation, creating a pro-angiogenic and anti-apoptotic microenvironment conducive to cancer development (27, 28).

In recent years, several novel molecular markers with prognostic value have been identified, including those from genomics, transcriptomics, epigenetics, proteomics, metabolomics, and radiomics. However, these approaches require high-quality samples and are costly, limiting their feasibility for routine clinical application (29–31). Additionally, although some biomarkers based on routine tests, such as the systemic immune inflammation index (SII), neutrophils-to-lymphocytes (NLR), platelets-to-lymphocytes (PLR), lymphocyte-to-monocyte ratio (LMR), and LMR-to-LDH ratio (LMR/LDH) are associated with DLBCL prognosis, they primarily focus on inflammatory parameters and overlook the significant role of metabolism in lymphoma prognosis, thus presenting certain limitations (32, 33). In this study, we found that the TyG index is an independent risk factor for DLBCL prognosis. Compared to existing prognostic indicators, the TyG index offers advantages such as accessibility, ease of implementation, and virtually zero cost. More importantly, the TyG index incorporates patients’ metabolic status, providing a more comprehensive reflection of patient characteristics and improving predictive accuracy.

While our findings support the potential of the TyG index as a valuable prognostic tool for DLBCL patients, this study has several limitations. Firstly, the retrospective nature of this study may introduce selection bias. In our research, patients were selected based on specific inclusion and exclusion criteria. Patients with certain comorbidities or those who did not complete four cycles of treatment were excluded, which might result in a non-representative sample of DLBCL patients. Secondly, missing data is a common issue in retrospective studies. While we included key prognostic variables for DLBCL, some critical parameters were unavailable in our dataset, potentially introducing residual confounding. Thirdly, as a single-center study, our results may be influenced by the specific patient population and treatment protocols of our institution. External validation through multicenter studies is warranted to confirm the broader applicability of our conclusions. Additionally, the relatively small sample size limits the statistical power of our analysis and may increase the risk of Type II errors. Larger-scale studies would be beneficial to confirm our conclusions. Lastly, we did not investigate the underlying mechanisms by which the TyG index is associated with poor prognosis in DLBCL patients. Further mechanistic studies are needed to explore the biological pathways linking the TyG index to disease progression and outcomes.

In summary, our study is the first to identify the TyG index as an independent prognostic factor for DLBCL. By integrating the TyG index with other key indicators, we developed a nomogram model that outperforms the traditional IPI score in predictive accuracy. Given its low cost, convenience, and practicality, the TyG index holds significant potential for clinical application. Additionally, regulating triglyceride and glucose levels—the main determinants of the TyG index—could offer promising therapeutic strategies for improving DLBCL prognosis. Nonetheless, further research is necessary to elucidate the specific mechanisms underlying this association.

## Data Availability

The original contributions presented in the study are included in the article/supplementary material. Further inquiries can be directed to the corresponding authors.
